# Appropriate Evaluation of Psychiatric Patients Highlighted by Creutzfeldt-Jakob Disease: A Case Report

**DOI:** 10.5811/cpcem.2020.7.47384

**Published:** 2020-09-03

**Authors:** Kathryn Bartlett, Kathleen E. Kane, Bryan G. Kane, Kevin R. Weaver, Gavin C. Barr

**Affiliations:** * Lehigh Valley Hospital and Health Network, Department of Emergency Medicine, Allentown, Pennsylvania; † University of South Florida Morsani College of Medicine, Department of Emergency Medicine, Tampa, Florida

**Keywords:** Cruetzfeldt-Jakob disease, psychiatric evaluation

## Abstract

**Introduction:**

Determination of medical stability for patients presenting with psychiatric complaints is common for emergency clinicians. A thorough history and physical examination is important.

**Case Report:**

A 53-year-old male presented to the emergency department (ED) with depression, suicidal ideation, and decline in activities of daily living over six months. While his initial neurologic examination was non-focal, subsequent re-evaluations demonstrated significant changes, and he was ultimately diagnosed with Creutzfeldt-Jakob disease.

**Conclusion:**

This case demonstrates how a detailed history of the present illness could have led to a more accurate and timely medical disposition from the ED.

## INTRODUCTION

The incidence of psychiatric concerns as chief complaints to the emergency department (ED) approaches 10%.^1^ Psychiatric patients have a higher incidence of morbidity and mortality than the general population; so it is important to use the ED encounter to ensure proper disposition and treatment.^2^ The history and physical examination are essential in the determination of medical stability prior to a psychiatric admission. Many medical conditions, including thyroid disorders, cerebrovascular accident, and dementia can have presentations that either manifest psychiatric symptoms or mimic psychiatric illness. This case of Creutzfeldt-Jakob disease (CJD) highlights several key points missed within the initial history and physical exam that, if identified in the ED, should have led to additional medical evaluation. While ultimately not changing the outcome, earlier identification would have expedited appropriate disposition of the patient. This case also highlights how an appreciation for several types of cognitive error can improve patient care.

## CASE REPORT

A 53-year-old, disheveled male presented to the ED with complaints of anxiety and depression with concomitant suicidal ideation. The patient noted worsening depression over the prior four to six months. His anxiety and depression led him to quit his job of 28 years as a bakery manager due to the inability to concentrate or follow directions. He attributed this to his worsening depression. The patient quit driving six days prior to presentation after an episode of forgetfulness caused him to run off the road. Review of systems revealed intermittent chest pain as well as decreased appetite and a 20-pound unintentional weight loss in the preceding several months, which the patient attributed to overwhelming anxiety. His wife also described multiple episodes in which he would blankly stare at nothing for seconds before returning to baseline.

The patient had a history of depression for many years prior to this presentation. However, the patient had never considered suicide or had difficulty with activities of daily life (ADL) prior to the last four months. His primary care physician documented normal physical examinations on an office visit within the prior month for depression and anxiety. His sertraline dose was increased by his primary care physician without improvement in symptoms.

In the ED, the patient’s physical examination was remarkable only for slow, tangential speech with repetitive answers. Initial laboratory testing, including toxicology, was unremarkable. Due to his chest discomfort at initial presentation, two serial troponins were obtained before deeming the patient medically stable. He signed a voluntary commitment for psychiatric treatment and was admitted to the behavioral health unit. On the inpatient psychiatric unit, the patient continued with depression and anxiety. While he no longer felt suicidal, he developed significant paranoia. His inattention worsened and he became increasingly disoriented. His speech, while slow, remained clear. However, despite several trials of antidepressants and antipsychotics, his symptoms persisted.

Due to the progression of his symptoms over several weeks, the patient was reevaluated medically. It was felt that his neurologic status was related to his psychiatric illness. Several days later, he developed brief episodes of an arm drop, as well as frequent staring episodes. At that point, neurologic consultation discovered deterioration when compared to previous neurologic examination. His speech was now solely confabulation. While strength and sensation remained intact, the patient now had mildly increased tone. Magnetic resonance imaging (MRI) of his brain, with and without contrast, a lumbar puncture (LP), and an electroencephalogram (EEG) were obtained.

MRI with diffusion-weighted imaging demonstrated high signal intensity/restricted diffusion in bilateral cerebral cortices and basal ganglia. As depicted in the image, the abnormality was more pronounced in the right hemisphere. Such findings can be seen with CJD, encephalitis, toxic/metabolic processes, and ischemic injury.

CPC-EM Capsule

What do we already know about this clinical entity?

*While guidelines for evaluation of psychiatric complaints exist, it is imperative to reevaluate patients with persistent progression of symptoms despite treatment*.

What makes this presentation of disease reportable?

*Nearly a third of patients with Creutzfeldt-Jakob Disease present with psychiatric complaints*.

What is the major learning point?

*Significant loss of daily life activities, neurologic findings on examination and changes in examinations should prompt a thorough medical work up*.

How might this improve emergency medicine practice?

*A thoughtful evaluation of patients presenting with psychiatric complaints will provide best practice patient care and ensure appropriate resource utilization*.

The EEG demonstrated slowing over the right hemisphere with anterior rhythmic slowing consistent with a diffuse encephalopathy. Cerebrospinal fluid (CSF) evaluation demonstrated large amounts of T-tau protein and was positive for CJD protein 14-3-3. These test results confirmed the diagnosis of CJD. The patient continued to decline cognitively; he was transitioned to hospice care and expired within one month of initial presentation to the ED.

## DISCUSSION

The history of present illness (HPI) and physical examination are the most important aspects of assessing medical stability of a psychiatric patient.^3^ Physicians must avoid anchoring, confirmation bias, and diagnosis momentum when evaluating patients presenting with psychiatric complaints. Anchoring bias is the failure to adjust decision-making process in the light of new information.^4^ Confirmation bias is the tendency to seek out information that supports the initial presumption, rather than to refute the initial thought.^4^ Diagnostic momentum exists when a label is placed on a patient or presentation, regardless of who places the label or how much information they have in support of that label.^4^ In this case, in addition to the HPI, the final diagnosis of CJD was made with MRI, EEG, and CSF analysis. CJD can be diagnosed as sporadic, familial, or iatrogenic.^5^ Sporadic is the most common type with typical age of onset at 60.^5^ The figure presents additional diagnostic detail regarding diagnostic criteria of CJD.^6^ A variant clinical presentation of CJD is characterized by psychiatric symptoms at an earlier stage with longer clinical deterioration and death at a younger age.^5^ Nearly one third of cases of variant CJD initially present with depression, emotional lability, behavioral changes, loss of appetite, and insomnia.^7^,^8^

In the case presented, rapid loss of ADLs and markedly worsening depression should have led to a broader differential diagnosis and expedited the patient’s disposition and care. The differential diagnosis for rapidly progressive dementia includes, but is not limited to, heavy metal toxicity, thyroid disorders, autoimmune disorders, vasculitis, sarcoidosis, viral or bacterial encephalopathies, and vitamin deficiencies, as well as CJD.^6^,^10^ While CJD will rarely be diagnosed in the ED, it is important to note the progressive decline presented in the HPI and to begin the appropriate medical evaluation.^6^,^8^

When evaluating a patient with rapidly progressive dementia, the work-up should include a complete blood count, comprehensive metabolic panel, thyroid function, and computed tomography of the brain. In consultation with neurology, other diagnostic studies may be warranted, including serology for neurosyphilis, paraneoplastic antibodies, or limbic encephalitis.^11^ In a prospective study of more than 500 patients with psychiatric illnesses, nearly 20% of the patients’ psychiatric illnesses could be attributed to medical ailments.^12^

MRI is more than 90% sensitive in the diagnosis of sporadic CJD.^7^ MRI images from patients with CJD often display patchy and extensive anomalies in more than one cortical region.^7^ Importantly, appropriate personal protective equipment (PPE) should be worn when performing an LP on someone suspected of having CJD. Ideally, disposable items should be used when collecting the CSF fluid; these should be incinerated as to avoid transmission of the prion protein.^13^ WHO guidelines on PPE for procedures provide a valuable resource for clinicians evaluating patients with suspected prion protein disease.^13^

CJD symptoms are caused by an abnormal accumulation and/or metabolism of prion proteins. The mutation in the prion protein results in the production of protease-resistant prion proteins, which cause nerve damage resulting in a variety of clinical presentations of the disease.^6^ For most patients diagnosed with CJD, rapid clinical progression from normal functioning to death occurs in approximately one year.^6^ CJD is ultimately fatal in all cases.^6^

## CONCLUSION

As the 2017 American College of Emergency Physicians clinical policy acknowledges, no laboratory or imaging studies are currently available to definitely and rapidly obviate all medical illnesses in psychiatric patients from the ED.^2^ Further supporting this is the 2017 American Association for Emergency Psychiatry comments that a thorough history and physical, including vital signs and a mental status examination, are of paramount importance when determining medical stability for patients with psychiatric illnesses.^14^ In the case presented, rapid loss of ADLs with behavioral changes should have increased the clinicians’ suspicion for a medical etiology. Further medical testing was indicated prior to determination of medical stability and admission to the behavioral health unit. This case highlights that recognition of concerning findings within the HPI and physical examination, as well as appreciation for minimization of cognitive bias, are important for identification of medical illnesses associated with psychiatric presentations and are necessary for appropriate disposition and treatment.

## Figures and Tables

**Figure:**
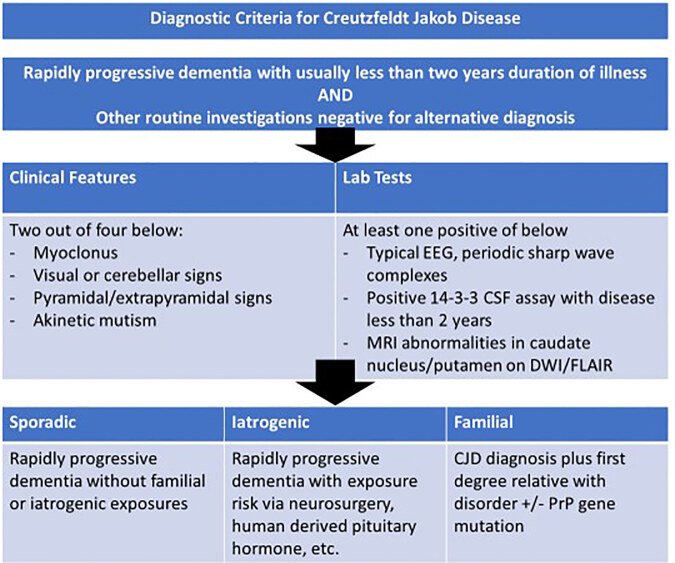
The diagnostic criteria for Creutzfeldt-Jakob disease.^9^ Amended from Centers for Disease Control and Prevention diagnostic criteria. *EEG*, electroencephalogram; *CSF*, cerebrospinal fluid; *MRI*, magnetic resonance imaging; *DWI*, diffusion-weighted imaging; *FLAIR*, fluid attenuated inversion recovery.

**Image:**
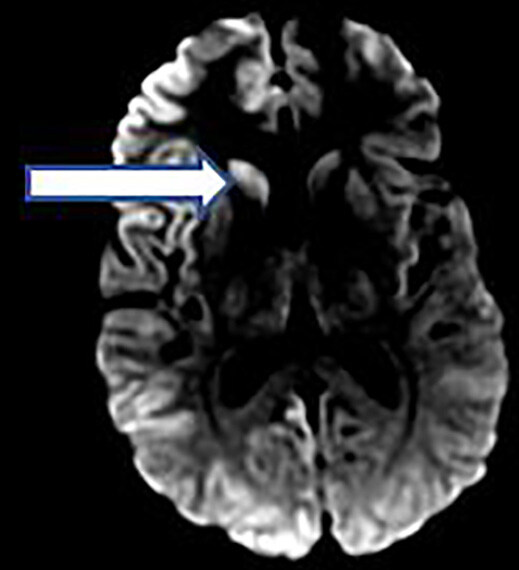
A single, magnetic resonance imaging slice of diffusion-weighted imaging of the patient’s brain. The image shows high-intensity signaling in the right basal ganglia, indicated by the arrow. The high-intensity signaling in the right basal ganglia is consistent with Creutzfeldt-Jacob disease.
